# Supplementary material for: HIVprotI: an integrated web based platform for prediction and design of HIV proteins inhibitors
Source: J Cheminform. 2018 Mar 9;10:12. doi: 10.1186/s13321-018-0266-y (PMC5845081; doi:10.1186/s13321-018-0266-y)
Supplement: Supplementary file 2 — Additional file 2. Source code of HIVProtI web server. [file 13321_2018_266_MOESM2_ESM.zip › HIVprotI_Source-code/hivprotI/weka-3-6-6/doc/weka/core/pmml/PMMLFactory.html]

PMMLFactory


---


|  |  |  |
| --- | --- | --- |
| **Overview** \| **Package** \| **Class** \| **Tree** \| **Deprecated** \| **Index** \| **Help** \| **Weka's home** | |  |
| **PREV CLASS**   **NEXT CLASS** | **FRAMES**    **NO FRAMES**     **All Classes** |  |
| SUMMARY: NESTED \| FIELD \| CONSTR \| METHOD | DETAIL: FIELD \| CONSTR \| METHOD |  |


---


## weka.core.pmml Class PMMLFactory

```
java.lang.Object
  weka.core.pmml.PMMLFactory
```

---

``` public class PMMLFactory extends java.lang.Object ```

This class is a factory class for reading/writing PMML models

**Version:**
:   $Revision: 5562 $

**Author:**
:   Mark Hall (mhall{[at]}pentaho{[dot]}com)

---

**Constructor Summary**

`PMMLFactory()`


| **Method Summary** | |
| --- | --- |
| `static java.lang.String` | `applyClassifier(PMMLModel model, Instances test)` |
| `static PMMLModel` | `getPMMLModel(java.io.File file)`             Read and return a PMML model. |
| `static PMMLModel` | `getPMMLModel(java.io.File file, Logger log)`             Read and return a PMML model. |
| `static PMMLModel` | `getPMMLModel(java.io.InputStream stream)`             Read and return a PMML model. |
| `static PMMLModel` | `getPMMLModel(java.io.InputStream stream, Logger log)`             Read and return a PMML model. |
| `static PMMLModel` | `getPMMLModel(java.lang.String filename)`             Read and return a PMML model. |
| `static PMMLModel` | `getPMMLModel(java.lang.String filename, Logger log)`             Read and return a PMML model. |
| `static void` | `main(java.lang.String[] args)` |
| `static void` | `serializePMMLModel(PMMLModel model, java.io.File file)`             Serialize a `PMMLModel` object that encapsulates a PMML model |
| `static void` | `serializePMMLModel(PMMLModel model, java.io.OutputStream stream)`             Serialize a `PMMLModel` object that encapsulates a PMML model |
| `static void` | `serializePMMLModel(PMMLModel model, java.lang.String filename)`             Serialize a `PMMLModel` object that encapsulates a PMML model |

**Methods inherited from class java.lang.Object**

`equals, getClass, hashCode, notify, notifyAll, toString, wait, wait, wait`

**Constructor Detail**

### PMMLFactory

```
public PMMLFactory()
```


**Method Detail**

### getPMMLModel

```
public static PMMLModel getPMMLModel(java.lang.String filename)
                              throws java.lang.Exception
```

:   Read and return a PMML model.

    :   **Parameters:**: `filename` - the name of the file to read from **Returns:**: a PMML model **Throws:**: `java.lang.Exception` - if there is a problem while reading the file

---


### getPMMLModel

```
public static PMMLModel getPMMLModel(java.io.File file)
                              throws java.lang.Exception
```

:   Read and return a PMML model.

    :   **Parameters:**: `file` - a `File` to read from **Returns:**: a PMML model **Throws:**: `java.lang.Exception` - if there is a problem while reading the file

---


### getPMMLModel

```
public static PMMLModel getPMMLModel(java.io.InputStream stream)
                              throws java.lang.Exception
```

:   Read and return a PMML model.

    :   **Parameters:**: `stream` - the `InputStream` to read from **Returns:**: a PMML model **Throws:**: `java.lang.Exception` - if there is a problem while reading from the stream

---


### getPMMLModel

```
public static PMMLModel getPMMLModel(java.lang.String filename,
                                     Logger log)
                              throws java.lang.Exception
```

:   Read and return a PMML model.

    :   **Parameters:**: `filename` - the name of the file to read from: `log` - the logging object to use (or null if none is to be used) **Returns:**: a PMML model **Throws:**: `java.lang.Exception` - if there is a problem while reading the file

---


### getPMMLModel

```
public static PMMLModel getPMMLModel(java.io.File file,
                                     Logger log)
                              throws java.lang.Exception
```

:   Read and return a PMML model.

    :   **Parameters:**: `file` - a `File` to read from: `log` - the logging object to use (or null if none is to be used) **Returns:**: a PMML model **Throws:**: `java.lang.Exception` - if there is a problem while reading the file

---


### getPMMLModel

```
public static PMMLModel getPMMLModel(java.io.InputStream stream,
                                     Logger log)
                              throws java.lang.Exception
```

:   Read and return a PMML model.

    :   **Parameters:**: `stream` - the `InputStream` to read from: `log` - the logging object to use (or null if none is to be used) **Throws:**: `java.lang.Exception` - if there is a problem while reading from the stream

---


### serializePMMLModel

```
public static void serializePMMLModel(PMMLModel model,
                                      java.lang.String filename)
                               throws java.lang.Exception
```

:   Serialize a `PMMLModel` object that encapsulates a PMML model

    :   **Parameters:**: `model` - the `PMMLModel` to serialize: `filename` - the name of the file to save to **Throws:**: `java.lang.Exception` - if something goes wrong during serialization

---


### serializePMMLModel

```
public static void serializePMMLModel(PMMLModel model,
                                      java.io.File file)
                               throws java.lang.Exception
```

:   Serialize a `PMMLModel` object that encapsulates a PMML model

    :   **Parameters:**: `model` - the `PMMLModel` to serialize: `file` - the `File` to save to **Throws:**: `java.lang.Exception` - if something goes wrong during serialization

---


### serializePMMLModel

```
public static void serializePMMLModel(PMMLModel model,
                                      java.io.OutputStream stream)
                               throws java.lang.Exception
```

:   Serialize a `PMMLModel` object that encapsulates a PMML model

    :   **Parameters:**: `model` - the `PMMLModel` to serialize: `stream` - the `OutputStream` to serialize to **Throws:**: `java.lang.Exception` - if something goes wrong during serialization

---


### applyClassifier

```
public static java.lang.String applyClassifier(PMMLModel model,
                                               Instances test)
                                        throws java.lang.Exception
```

:   **Throws:**: `java.lang.Exception`

---


### main

```
public static void main(java.lang.String[] args)
```


---


|  |  |  |
| --- | --- | --- |
| **Overview** \| **Package** \| **Class** \| **Tree** \| **Deprecated** \| **Index** \| **Help** \| **Weka's home** | |  |
| **PREV CLASS**   **NEXT CLASS** | **FRAMES**    **NO FRAMES**     **All Classes** |  |
| SUMMARY: NESTED \| FIELD \| CONSTR \| METHOD | DETAIL: FIELD \| CONSTR \| METHOD |  |


---
